# Fluoroquinolone and Other Antimicrobial Resistance in Invasive Pneumococci, Hong Kong, 1995–2001

**DOI:** 10.3201/eid1007.030612

**Published:** 2004-07

**Authors:** Pak-Leung Ho, Tak-Lun Que, Susan S. Chiu, Raymond W. H. Yung, Tak-Keung Ng, Dominic N.C. Tsang, Wing-Hong Seto, Yu-Lung Lau

**Affiliations:** * Queen Mary Hospital, The University of Hong Kong, Hong Kong Special Administrative Region (SAR), China;; † Tuen Mun Hospital, Hong Kong SAR, China;; ‡ Pamela Youde Nethersole Eastern Hospital, Hong Kong SAR, China;; § Princess Margaret Hospital, Hong Kong, SAR, China;; ¶ Queen Elizabeth Hospital, Hong Kong SAR, China

**Keywords:** *Streptococcus pneumoniae*, drug resistance, microbial, anti-infective agents, fluoroquinolone, multilocus sequence typing, research

## Abstract

We determined the susceptibilities of 265 invasive isolates of pneumococci obtained during 1995 to 2001 in Hong Kong to 11 antimicrobial agents and their serotypes. Overall, 62.6% isolates were susceptible to penicillin, 20% were intermediately resistant, and 17.4% were resistant. The overall prevalence of levofloxacin resistance (MIC >8 µg/mL) was 3.8% but increased to 15.2% among the penicillin-resistant isolates. All levofloxacin-resistant isolates were clonally related; had reduced susceptibility to penicillin, cefotaxime, and clarithromycin; and were derived from adults >50 years of age. Of the penicillin-nonsusceptible pneumococci, 90% from children <5 years of age and 54.8% from persons of all ages were of serotypes that are included in the 7-valent pneumococcal conjugate vaccine; 93.5% from children <5 years of age and 93% from persons of all ages were of serotypes that are included in the 23-valent polysaccharide vaccine.

## Abstract 2

Fluoroquinolone resistance among invasive pneumococci in Hong Kong was high and a result of clonal expansion and spread.

The emergence of antimicrobial resistance in *Streptococcus pneumoniae* worldwide is an important public health issue because this organism is the leading cause of many infections, particularly community-acquired pneumonia. In many countries, rates of resistance for penicillin, macrolides, and tetracyclines have reached levels of 30% to 40% or higher and are increasing. In recent years, the emergence of fluoroquinolone resistance is being increasingly recognized among multidrug-resistant strains of *S. pneumoniae*. Hong Kong, Ireland, Canada, and Spain have reported increasing rates of fluoroquinolone resistance among *S. pneumoniae* (*1**–**3*). So far, reports on fluoroquinolone resistance in *S. pneumoniae* have predominantly involved respiratory tract isolates, and whether this type of resistance is emerging among the invasive isolates is unknown. In this study, we evaluated the comparative activities of five fluoroquinolones against invasive isolates of *S. pneumoniae* from Hong Kong that were collected during a period when fluoroquinolone resistance had increased rapidly among noninvasive respiratory isolates.

## Materials and Methods

### Bacterial Isolates

Stored isolates of *S. pneumoniae* were obtained from the blood and cerebrospinal fluid (CSF) of patients admitted to five hospitals in Hong Kong during 1995 to 2001. These five hospitals were chosen because they represent the same sentinel network that participated in an earlier study conducted by the same group (*4*). One hospital did not store the isolates and thus was not included in the present study. The following numbers of isolates were obtained from each of the hospitals: A (140 isolates from 1995 to 2001), B (22 isolates from 1996 to 2001), C (34 isolates from 1997 to 2001), D (64 isolates from 1998 to 2001), and E (5 isolates from 2001). All are public hospitals that provide acute patient care, including all the major specialties. Hospital A is a university teaching hospital with a bone marrow transplant unit, and the others are regional hospitals. Hospitals A and B are located in the same region, and they together serve a population of approximately 1.4 million. Hospitals C, D, and E serve populations of 0.6, 1, and 0.4 million, respectively. Thus, this network together serves approximately 53% of the 6.5 million population in Hong Kong. Isolates included in this study represented all the invasive pneumococcal case-patients with a positive blood or CSF culture in the stated periods. All invasive isolates were tested for antimicrobial susceptibility. Only one isolate from the same patient episode of infection was included. Three patients had two episodes of infections separated by intervals of 3 months to 2 years. All isolates were subcultured and reidentified by considering the following characteristics: colony morphologic features, Gram stain results, optochin susceptibility, and bile solubility. Isolates were stored at –20°C until they were tested in batches.

### Antimicrobial Agents and Susceptibility Testing

E-test strips of penicillin, amoxycillin (as amoxycillin-clavulanate 2:1), cefotaxime, cefepime, clarithromycin, vancomycin, ciprofloxacin, levofloxacin, sparfloxacin, gatifloxacin, and moxifloxacin were purchased from AB Biodisk, Solna, Sweden. E-test MICs were determined following the manufacturer's instructions. All susceptibility testing was conducted in a single laboratory at the University of Hong Kong. Test inocula were prepared from pneumococcal colonies grown on sheep blood agar that had been incubated for 20 to 24 h in 5% CO_2_. Colonies were suspended in 0.9% saline to obtain a suspension equivalent to a 0.5 McFarland standard of turbidity. From this suspension, E-tests were performed on Mueller-Hinton agar with 5% sheep blood (BBL, Becton Dickinson Microbiology Systems, Cockeysville, MD). The plates were incubated at 35°C in 5% CO_2_ for 20 h to 24 h. MICs falling between two marks on the E-test strip were rounded up to the next higher twofold dilution, as recommended in the instructions. For all MIC determinations, the bacterial inocula were validated by back titration in 10% of the tests to ensure the desired inoculum density. Quality control strains (*S. pneumoniae* ATCC 49619, *Staphylococcus aureus* ATCC 29213, and *Escherichia coli* ATCC 25922) were included with each run. Results were interpreted according to published breakpoints of the National Committee for Clinical Laboratory Standards (*5*). The term nonsusceptible was used to denote both intermediate and resistant isolates. For ciprofloxacin, the criteria were susceptible, <2 µg/mL; resistant, >4 µg/mL.

### Typing of Isolates

All isolates were serotyped by the quellung reaction (*6*) with sera of various levels of reactivity from the Statens Seruminstitut (Copenhagen, Denmark). The subset of 11 isolates with resistance to ciprofloxacin was examined further by multilocus sequence typing (MLST) and by *Hinf*I restriction analysis of their *pbp* 2b and 2x genes (*7*). The well-defined Spanish clones of serotypes 23F and 6B (SP264 ATCC 700669 and GM17 ATCC 700670, respectively) and a strain representative of the fluoroquinolone-resistant variant Hong Kong^23F^-1 clone were used as controls (*4*).

### Polymerase Chain Reaction (PCR) and DNA Sequencing

The quinolone resistance–determining regions of gyrA, gyrB, parC, and parE were amplified by using primers described previously (*8*). Nucleotide sequencing was performed by the ByeDye dideoxynucleotide chain termination method (Applied Biosystems, Hong Kong). The sequences of both strands of the amplicons were determined.

### Statistical Analysis

Chi-square or Fisher exact test was used for statistical analysis. A p value of <0.05 was considered significant.

## Results

### Emergence of Fluoroquinolone Resistance among Multidrug-resistant Isolates

**Figure 1:**
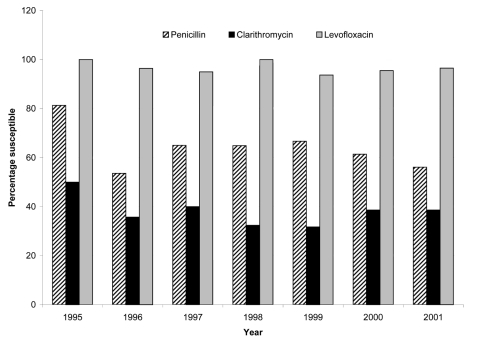
Susceptibility rates for 265 invasive *Streptococcus pneumoniae* in Hong Kong by year, 1995–2001.

The number of isolates obtained from different age groups was as follows: <2 years (n = 48); 2–5 years (n = 40); 6–17 years (n = 14); 18–49 years (n = 30); 50–64 (n = 27), and >65 years (n = 106). Of the isolates, 256 (96.6%) were from blood, 6 from CSF, and 3 from brain abscess. The susceptibilities of the 265 pneumococcal isolates to 11 antimicrobial agents are summarized in Table 1. The annual susceptibility rates for penicillin, clarithromycin, and levofloxacin are shown in Figure 1. Overall, 166 (62.6%) were penicillin-susceptible, 53 (20%) were penicillin-intermediate, and 46 (17.3%) were penicillin-resistant. Rates of penicillin nonsusceptibility (MIC > 0.06 µg/mL) do not differ significantly in the five hospitals (p = 0.1): 35.7% (50 of 140) for laboratory A, 50% (11 of 22) for laboratory B, 32.4% (11 of 34) for laboratory C, 37.5% (24 of 64) for laboratory D, and 60% (3 of 5) for laboratory E.

**Table 1:** MICs of 11 antimicrobial agents for *Streptococcus pneumoniae* isolates based on susceptibility to penicillin^a,b^

Antimicrobial agent and penicillin susceptibility status	MIC (µg/mL)				% of Isolates		
	Range	50%	90%	Mode	S	I	R
Penicillin							
All	0.008–4	0.032	2	0.016	62.6	20.0	17.4
Pen-S	0.008–0.064	0.016	0.032	0.016	100	0.0	0.0
Pen-I	0.125–1	1	1	1	0.0	100	0.0
Pen-R	2–4	2	2.0	2	0.0	0.0	100
Amoxicillin							
All	0.016–4	0.032	2	0.016	99.6	0.4	0.0
Pen-S	0.016–0.125	0.016	0.032	0.016	100.0	0.0	0.0
Pen-I	0.032–2	1	2	1	100.0	0.0	0.0
Pen-R	0.5–4	2	2	2	97.8	2.2	0.0
Cefotaxime							
All	0.016–4	0.032	1	0.016	97.0	2.6	0.4
Pen-S	0.016–0.125	0.016	0.032	0.016	100.0	0.0	0.0
Pen-I	0.032–2	1	1	1	100.0	0.0	0.0
Pen-R	0.5–4	1	2	1	82.6	15.2	2.2
Cefepime							
All	0.016–4	0.064	2	0.064	76.2	21.9	1.9
Pen-S	0.016–0.25	0.064	0.125	0.064	100.0	0.0	0.0
Pen-I	0.032–1	1	2	2	66.0	34.0	0.0
Pen-R	1–4	2	2	2	2.2	87	10.9
Clarithromycin							
All	0.032–256	4	256	256	36.6	0.4	63.0
Pen-S	0.032–256	0.125	256	0.125	56.0	0.0	44.0
Pen-I	0.064–256	256	256	256	7.5	1.9	90.6
Pen-R	2–256	4	256	2	0.0	0.0	100.0
Vancomycin							
All	0.25–1	0.5	0.5	0.5	100.0	0.0	0.0
Pen-S	0.25–1	0.5	0.5	0.5	100.0	0.0	0.0
Pen-I	0.25–1	0.5	0.5	0.5	100.0	0.0	0.0
Pen-R	0.25–1	0.5	1	0.5	100.0	0.0	0.0
Ciprofloxacin							
All	0.25–32	1	1	1	95.8	–	4.2
Pen-S	0.25–4	1	1	1	99.4	–	0.6
Pen-I	0.25–32	1	1	1	94.3	–	5.7
Pen-R	0.5–32	1	32	1	84.8	–	15.2
Levofloxacin							
All	0.125–32	1	1	1	96.2	0.0	3.8
Pen-S	0.125–2	1	1	1	100.0	0.0	0.0
Pen-I	0.125–32	1	1	1	94.3	0.0	5.7
Pen-R	0.5–32	1	32	1	84.8	0.0	15.2
Sparfloxacin							
All	0.125–32	0.25	0.5	0.5	95.8	0.8	3.2
Pen-S	0.125–1	0.25	0.5	0.5	99.4	0.6	0.0
Pen-I	0.125–32	0.25	0.5	0.5	94.3	0.0	5.7
Pen-R	0.25–32	0.5	32	0.5	84.8	2.2	13.0
Gatifloxacin							
All	0.064–16	0.25	0.25	0.25	96.2	0.8	3.0
Pen-S	0.064–1	0.25	0.25	0.25	100.0	0.0	0.0
Pen-I	0.125–8	0.25	0.25	0.25	94.3	1.9	3.8
Pen-R	0.125–16	0.25	8	0.25	84.8	2.2	13.0
Moxifloxacin							
All	0.064–8	0.125	0.25	0.125	97.0	0.8	2.3
Pen-S	0.064–1	0.125	0.25	0.125	100.0	0.0	0.0
Pen-I	0.064–4	0.125	0.25	0.125	96.2	1.9	1.9
Pen-R	0.125–8	0.125	4	0.125	87.0	2.2	10.9

^a^N = 265; a total of 166 isolates were penicillin-susceptible (Pen-S), 53 were penicillin-intermediate (Pen-I), and 46 were penicillin-resistant (Pen-R). ^b^50% and 90%, MIC_50_ and MIC_90_, respectively.

In children (ages <12 years), the rate of penicillin nonsusceptibility was significantly higher than that in adults (>13 years of age) (48% vs. 30.9%, respectively; p = 0.005). In general, MICs of penicillin were identical or within one dilution difference of that of amoxicillin. For these two penicillins, the MIC_50_, MIC_90_, and mode MIC values were identical. On the other hand, MICs of cefotaxime were generally one dilution lower than that of cefepime. High MICs of penicillin (4 µg/mL) and cefotaxime (4 µg/mL) were found in 2 (0.8%) and 1 (0.4%) of 265 isolates, respectively.

Of the 265 isolates, 97 (36.6%) were clarithromycin-susceptible, 1 (0.4%) was clarithromycin-intermediate, and 167 (63%) were clarithromycin-resistant. In most isolates, the resistance was of a high level type. Among 168 clarithromycin-nonsusceptible isolates, 102 (60.7%) had MIC >32 µg/mL, and 78 (46.4%) had MIC >256 µg/mL. Again, clarithromycin resistance rate was higher among children than adults (83 [83%] of 100 vs. 85 [51.5%] of 165, respectively; p < 0.001). The clarithromycin-nonsusceptibility rates in penicillin-susceptible and nonsusceptible isolates were 73 (44%) of 166 and 95 (95.9%) of 99, respectively (p < 0.001).

Overall, 10 (3.8%) of 265 isolates were resistant to levofloxacin. The levofloxacin-resistance rate increased to 15.2% among the penicillin-resistant pneumococcal isolates and was 7.5% among isolates derived from persons >50 years of age. One penicillin-susceptible isolate (S1D3) had a ciprofloxacin MIC of 4 µg/mL. This isolate remained susceptible to levofloxacin. The 10 resistant isolates had a levofloxacin MIC from 16 µg/mL to 32 µg/mL; 8 of these were resistant to gatifloxacin (MIC range, 4–16 µg/mL), and 6 were resistant to moxifloxacin (MIC range, 4–8 µg/mL). All levofloxacin-resistant isolates were also either intermediately resistant or resistant to penicillin (MIC range, 1–4 µg/mL) and clarithromycin (MIC range, 2–>256 µg/mL). All levofloxacin-resistant isolates were from adults (one from the 50-to 64-year age group and nine from >65-year group). Seven were nosocomial infections (having onset >2 days after admission), and three were community-acquired infections. For the levofloxacin-susceptible isolates, the rank order of potency (MIC_50_/MIC_90_) was as follows: moxifloxacin (0.125/0.25) > gatifloxacin (0.25/0.25) > sparfloxacin (0.25/0.5) > levofloxacin (1/1) = ciprofloxacin (1/1).

### Serotype Distribution of Isolates and Resistance Patterns

Of 265 isolates, 8 isolates could not be typed; 34 different serotypes were identified among the remaining isolates. The serotype distribution of the isolates according to age group of patients and penicillin resistance is shown in Table 2. Serotype 14 was the most common serotype (24.5%). Four serotypes (6B, 14, 19F, and 23F) accounted for 92.9% of all penicillin-nonsusceptble isolates and 84.5% of all clarithromycin-nonsusceptible isolates. The capsular serotypes of the levofloxacin-resistant isolates were 14 (n = 4), 19F (n = 2), and 23F (n = 4). Serotypes included in 7-valent pneumococcal conjugate vaccine formulations (4, 6B, 9V, 14, 18C, 19F, and 23F) comprised 90.4% and 90.6% of penicillin- and clarithromycin-nonsusceptible strains isolated from persons with age <5 years, respectively. Coverage of the 7-valent conjugate vaccine for all isolates from young children (<5 years of age) was 89.7% (79/88). Serotypes included in the 23-valent pneumococcal polysaccharide vaccine accounted for 92.9% of penicillin-nonsusceptible and 91.1% of clarithromycin-nonsusceptible isolates for all ages, respectively.

**Table 2:** Distribution of pneumococcal capsular types according to age group of patient and penicillin resistance, 1995–2001

Serotype^a^	All ages		Age <5 years	
	No. of isolates	Penicillin-resistant (%)	No. of isolates	Penicillin-resistant (%)
*14*	65	32.3	31	25.8
*23F*	46	82.6	15	80
*6B*	26	69.2	18	72.2
3	24	0	1	0
*19F*	19	78.9	9	55.6
*18C*	12	0	4	0
*9V*	8	0	2	0
*4*	7	0	0	0
All others	58	12.1	8	50
Total	265	37.4	88	47.7

^a^MIC >0.12 µg/mL. Serotypes included in 7-valent pneumococcal conjugate vaccine formulations included 4, 6B, 9V, 14, 18C, 19F, and 23F (in italics).

### Molecular Analysis of Fluoroquinolone-resistant Isolates

**Figure 2:**
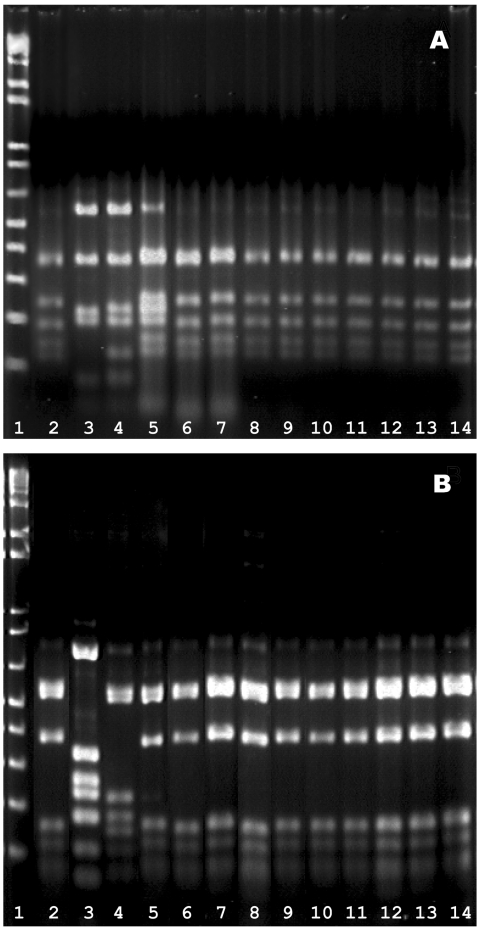
*Hinf*I fingerprints of the *pbp* genes. A, *pbp2b* profiles. Lanes 1, marker; 2, Spain^23F-1^ clone (SP264, ATCC 700669); 3, a ciprofloxacin-resistant, levofloxacin-susceptible strain S1D3; 4, Spain^6B^ clone (GM17, ATCC 700670); lane 5–14, 10 isolates of levofloxacin resistant pneumococci (S3F7, S2H9, S1B7, S1B9, S1D5, S2D6, S1D2, S2F3, 186G1, and 216D2, respectively); B, *pbp*2x profiles. The lanes were arranged in the same sequence as in A.

Molecular analysis of the 11 fluoroquinolone-resistant isolates is summarized in Table 3. Analysis by MLST showed that a single allelic profile (4-4-2-4-4-1-1) or sequence type (ST 81) was shared by all 10 levofloxacin-resistant isolates. Fingerprint patterns after *Hinf*I digestion of the amplified *pbp* 2x and 2b genes are shown in the Figure 2. One fingerprint pattern for *pbp* 2b was shared by nine levofloxacin-resistant isolates. The remaining levofloxacin-resistant isolate has a pattern that differed from the major pattern by one band. A single fingerprint pattern for *pbp* 2x was shared by all 10 levofloxacin-resistant isolates. Both *pbp* 2b and 2x fingerprint patterns among the levofloxacin-resistant isolates were indistinguishable from that displayed by the Spain^23F-1^ clone. The remaining ciprofloxacin-resistant, levofloxacin-susceptible strain had distinct *pbp* 2b and 2x fingerprint patterns. Furthermore, the 10 isolates all had similar pattern of mutations in *gyrA*, *parC*, and *parE* genes. In GyrA, all 10 isolates had a S81F or Y substitution. In ParC, the 10 isolates had at least one amino acid substitution, and 6 isolates had two substitutions, an S79F plus K137N pair. In ParE, one isolate had no substitution. Five isolates had one substitution (I460V), and four had two substitutions (1460V plus D435 or E474K pair). The remaining ciprofloxacin-resistant, levofloxacin-susceptible isolate (S1D3) had a distinct MLST pattern and PBP 2B and 2X gene profiles. This isolate had one substitution in each of ParC and ParE. No strains had substitutions in GyrB.

**Table 3:** Characteristics of 11 strains of *Streptococcus pneumoniae* with reduced susceptibility to ciprofloxacin

Strain	Y	Source	Serotype^a^	MLST profile^a^	MIC (µg/mL)^b^				Mutation in QRDR of^c,d^		
					CIP	LVX	GAT	MO	GyrA	ParC	ParE
S3F7	1996	A	23F	4-4-2-4-4-1-1	32	32	2	1	S81F	K137N	E474K, I460V
S2H9	1997	A	23F	4-4-2-4-4-1-1	32	32	4	2	S81Y	K137N	D435N, I460V
S1B7	1999	B	23F	4-4-2-4-4-1-1	32	32	8	4	S81F	S79F, K137N	I460V
S1B9	1999	B	23F	4-4-2-4-4-1-1	32	32	16	4	S81F	S79F, K137N	I460V
S1D5	1999	C	19F	4-4-2-4-4-1-1	32	32	8	4	S81F	S79F, K137N	I460V
S2D6	1999	A	14	4-4-2-4-4-1-1	32	32	8	4	S81F	S79F, K137N	I460V
S1D2	2000	C	19F	4-4-2-4-4-1-1	32	32	8	8	S81F	S79F, K137N	–
S1D3	2000	C	4	8-8-8-1-17-1-18	4	2	0.5	0.25	–	K137N	I460V
S2F3	2000	A	14	4-4-2-4-4-1-1	32	32	4	2	S81F	K137N	D435N, I460V
186G1	2001	A	23F	4-4-2-4-4-1-1	32	16	2	0.5	S81F	K137N	E474K, I460V
216D2	2001	A	14	4-4-2-4-4-1-1	32	32	4	4	S81F	S79F, K137N	I460V

^a^MLST, multilocus sequence typing. Number refers to allelle of *aroE*, *gdh*, *gki*, *recP*, *spi*, *xpt*, and *ddiA* genes, respectively. ^b^CIP, ciprofloxacin; LVX, levofloxacin; GAT, gatifloxacin; MO, moxifloxacin. ^c^No strains had mutations in GyrB sequence, QRDR, quinolone resistance–determining region. ^d^*S. pneumoniae* numbering.

## Discussion

This study showed that fluoroquinolone resistance among pneumococci that cause invasive infections is emerging. Our finding of a 3.8% resistance rate for levofloxacin is among the highest ever reported in the world and could be attributed in part to suboptimal use of the fluoroquinolones (*9*). In a case-control study, we have previously shown that chronic obstructive airway disease, nosocomial infection, nursing home residence, and exposure to lesser potent fluoroquinolones were independently associated with fluoroquinolone-resistant *Streptococcus pneumoniae* (*9*). Elsewhere, levofloxacin resistance among the invasive pneumococcal isolates was still rare at <1% (*10**–**12*). In the United States, Jorgensen et al. recently reported that fluoroquinolone-resistant pneumococci could be also emerging in some of the Active Bacterial Core Surveillance Areas (ABCs). Of 538 invasive pneumococci collected from 1998 to 2000 from California, 3.2% had ciprofloxacin MIC of >4 µg/mL (*13*). In general, antimicrobial resistance among the pneumococci occurred more frequently among respiratory tract isolates than blood isolates (*2*). In our previous studies, the fluoroquinolone resistance rate among the respiratory isolates was 5.5% and 13% in 1998 and 2000, respectively (*3**,**4*).

Our findings show that invasive pneumococci with fluoroquinolone resistance in this locality were related to the multidrug-resistant Spanish 23F clone. Three different serotypes were identified among the 10 clonally related levofloxacin-resistant isolates, indicating that this clone is evolving by horizontal transfer of the capsular genes. Elsewhere, early evidence suggested that epidemic clones could be playing a role in dissemination of fluoroquinolone resistance. In an analysis of 29 fluoroquinolone-resistant pneumococci, McGee et al. reported that 6 isolates from Ireland and 1 from France were indistinguishable from the Spain^9V^-3 clone (*14*). In Birmingham, George et al. recently reported that two fluoroquinolone-resistant variants closely related to the widely distributed penicillin-resistant Spanish^9V^-3 clone were emerging (*15*). Furthermore, Alou et al. report that 30% of 82 pneumococci with reduced susceptibility to ciprofloxacin from 20 hospitals in Spain belonged to two internationally spread clones: France^9V^-3 and Spain^23F^-1 (*16*). The emergence of fluoroquinolone resistance among the internationally distributed *S. pneumoniae* clones is of concern. The Hong Kong experience is an example of how resistance to the fluoroquinolones could evolve rapidly in pneumococci as a result of clonal expansion.

This study found a high rate of macrolide resistance among the invasive pneumococcal isolates, as was reported for noninvasive isolates (*4*). This circumstance is likely related to the high local use of macrolides and the dissemination of several drug-resistant clones (*17*). Among invasive isolates, our figure was similar to that reported in Taiwan (72%) but was higher than those reported from the United States (20.4%), Canada (14.8%), or Germany (15.3%) (*18**–**21*). Despite early skepticism, increasing evidence shows that in vitro macrolide resistance does result in clinical and microbiologic failures in systemic pneumococcal infection (*22**,**23*). Hence, in the empirical treatment of community-acquired pneumonia, our findings imply that monotherapy with a macrolide is not appropriate in this region.

Our data show that 90% or more of the resistant pneumococci that cause invasive infections in persons of all ages belonged to serotypes that are included in the 23-valent pneumococcal polysaccharide vaccine as well as the 7-valent conjugate vaccine. The 7-valent conjugate vaccine is indicated in young children and is highly effective in preventing vaccine serotype-related invasive diseases (*24*). In the United States, the 7-valent conjugate vaccine was added to the routine schedule in 2000. According to Whitney et al. (*25*), after the conjugate vaccine was introduced, the rate of invasive disease caused by vaccine and vaccine-related serotypes has markedly declined. The rate of disease caused by strains that were not susceptible to penicillin was 35% lower in 2001 than in 1999. The rate of disease in adults also declined (*25*). From the results of trials reported so far, the vaccine will likely also reduce carriage of vaccine types of pneumococci (*26**,**27*). Hence, resistant pneumococci might diminish as the vaccine becomes more widely available (*25**,**28*). The effectiveness of the 23-valent polysaccharide vaccine has not been as dramatic. In older adults, vaccination with the polysaccharide vaccine effectively reduced the rate of bacteremia but not that of nonbacteremic pneumonia (*29*). The use of the 23-valent pneumococcal vaccine in Hong Kong is low (with estimated coverage of <10% for those >65 years). While data on the efficacy of the 23-valent pneumococcal vaccine are considered insufficient in patients with chronic obstructive pulmonary disease (*30*), the benefits of vaccinating elderly people with the 23-valent pneumococcal vaccine are clear (*31*). In view of the findings from this and our previous study that the elderly and persons with chronic obstructive pulmonary disease are at high risk of developing infection by fluoroquinolone-resistant *S. pneumoniae*, we believe both patient groups in this locality should receive pneumococcal vaccine.

In conclusion, this study reported high rates of fluoroquinolone resistance among multidrug-resistant strains of pneumococci that cause invasive infections among older adults in Hong Kong. Our experience leads us to call for a more prudent use of fluoroquinolones in all clinical settings. Since carriage of pneumococci is common, collateral exposure could occur anytime a person is treated with fluoroquinolones for any infection, including skin, soft tissue, or urinary tract infections. In patients with chronic obstructive pulmonary disease, high-density colonization of the airway is common, which could explain why these patients are at high risk for fluoroquinolone-resistant pneumococci (*9*). In young children, high numbers of pneumococci are frequently found in the nasopharynx. If fluoroquinolone use is extended from adults to children, who are frequently colonized by the antimicrobial-resistant serotypes, transmission and spread of fluoroquinolone-resistant strains would occur rapidly in the community (*32*). For pneumonia, all evidence so far indicates that infection caused by pneumococci intermediately resistant to penicillin (MIC <1 µg/L or 2 µg/mL) should respond well to a penicillin given in appropriate doses. In view of this and the emergence of fluoroquinolone resistance in both noninvasive and invasive isolates in this locality, we believe that fluoroquinolones should not be used as first-line treatment in community-acquired pneumonia. In local guidelines, amoxicillin-clavulanate or the combination of amoxicillin and a new macrolide are the recommended first-line drugs for empirical treatment of community-acquired pneumonia in the outpatient setting. As pneumococcal infections become increasing difficult to treat, public health authorities should give priority to pneumococcal vaccination of persons at high risk of acquiring infections by the resistant pneumococci. Since substantial changes can occur in a short period, fluoroquinolone-resistant isolates must be monitored and tracked as part of ongoing and routine pneumococcal surveillance.
